# Pediatric Traumatic Brain Injury in the United States: Rural-Urban Disparities and Considerations

**DOI:** 10.3390/brainsci10030135

**Published:** 2020-02-28

**Authors:** John K. Yue, Pavan S. Upadhyayula, Lauro N. Avalos, Tene A. Cage

**Affiliations:** 1 Department of Neurological Surgery, University of California San Francisco, San Francisco, CA 94143, USA; lauro.avalos@ucsf.edu; 2 Brain and Spinal Injury Center, Zuckerberg San Francisco General Hospital, San Francisco, CA 94110, USA; 3 Department of Neurological Surgery, Columbia University of Physicians and Surgeons, New York, NY 10027, USA; psupadhy@ucsd.edu; 4 Department of Neurological Surgery, Stanford University, Stanford, CA 94305, USA; tenecage@stanford.edu

**Keywords:** concussion, cost, epidemiology, health disparity, rural, traumatic brain injury, underserved

## Abstract

Introduction: Traumatic brain injury (TBI) remains a primary cause of pediatric morbidity. The improved characterization of healthcare disparities for pediatric TBI in United States (U.S.) rural communities is needed to advance care. Methods: The PubMed database was queried using keywords ((“brain/head trauma” OR “brain/head injury”) AND “rural/underserved” AND “pediatric/child”). All qualifying articles focusing on rural pediatric TBI, including the subtopics epidemiology (*N* = 3), intervention/healthcare cost (*N* = 6), and prevention (*N* = 1), were reviewed. Results: Rural pediatric TBIs were more likely to have increased trauma and head injury severity, with higher-velocity mechanisms (e.g., motor vehicle collisions). Rural patients were at risk of delays in care due to protracted transport times, inclement weather, and mis-triage to non-trauma centers. They were also more likely than urban patients to be unnecessarily transferred to another hospital, incurring greater costs. In general, rural centers had decreased access to mental health and/or specialist care, while the average healthcare costs were greater. Prevention efforts, such as mandating bicycle helmet use through education by the police department, showed improved compliance in children aged 5–12 years. Conclusions: U.S. rural pediatric patients are at higher risk of dangerous injury mechanisms, trauma severity, and TBI severity compared to urban. The barriers to care include protracted transport times, transfer to less-resourced centers, increased healthcare costs, missing data, and decreased access to mental health and/or specialty care during hospitalization and follow-up. Preventative efforts can be successful and will require an improved multidisciplinary awareness and education.

## 1. Introduction

Traumatic brain injury (TBI) remains a leading cause of mortality and morbidity in the United States (U.S.). The majority of TBI is classified as mild TBI (mTBI, 80–90%), with only 5–10% of TBI categorized as moderate and severe [1,2]. Young children aged 0–4 and older adolescents aged 15–19 have the highest incidence of TBI across the population groups, highlighting the importance of understanding the TBI diagnosis and treatment in these patient populations [3]. 

The pediatric population is uniquely vulnerable, with over 50,000 annual hospitalized cases [4]. TBI can be devastating to the developing central nervous system and lead to protracted care and poorer outcomes in settings with suboptimal resources and/or triage. Children aged 0–4 experience the highest mortality rates across age groups, with unintentional injuries, including motor vehicle accidents, accounting for the highest percentage of non-perinatal mortality [5]. Prior to recent evidence, mTBI, as defined by the Glasgow Coma Scale (GCS) score 13–15, was thought to have insignificant deficits that would resolve over time, but it is now understood that even “milder” head injury can have functional, cognitive, and psychiatric sequelae lasting months to years post-injury [6,7].

Rural patients often do not have access to the same level of resources as their urban counterparts, which can impact long term outcomes. Pediatric tertiary care and Level I trauma centers are available and accessible in most urban cities in the U.S. [8]. A study in rural Idaho estimated that up to 50% of rural patients live >60 miles and 25% live >90 miles from a tertiary care center [9]. Medicare data across rural areas in five U.S. states showed that rural patients needed to travel two to three times further to reach medical and surgical specialists compared to urban patients [10]. Rural children often experience lapses in adequate healthcare, delays in service due to longer transportation times, and decreased access to physicians that are trained to appropriately treat specific complex health conditions [11,12].

Acute and rehabilitation care are critical to improving TBI outcomes. Severe TBI occurring in rural areas often requires transfer to tertiary care centers, with lower access to operative management availability [13]. Though mTBI evaluation and care paradigms are available in rural emergency departments (EDs), significant functional deficits remain in certain subpopulations [14,15]. Rural patients are at an increased risk of inadequate initial evaluation and lack of proper follow-up that affects even urban centers. Disparities in healthcare costs associated with triage, transport, treatment, and rehabilitation are also important to consider.

An improved characterization of healthcare disparities in the U.S. between rural/urban settings for pediatric TBI treatment is needed to advance care. The current review synthesizes the available primary literature to date on the topic, across subtopics of epidemiology, intervention and healthcare costs, and prevention for U.S. pediatric TBI.

## 2. Methods

### 2.1. Study Selection

The National Library of Medicine PubMed database was queried for primary literature using the search term: ((pediatric [title/abstract] *OR* child [title/abstract]) *AND* (brain injury [title/abstract] OR TBI [title/abstract] *OR* head injury [title/abstract] *OR* brain trauma [title/abstract] *OR* head trauma [title/abstract] *OR* concussion [title/abstract]) *AND* (rural [title/abstract] *OR* underserved [title/abstract])) in the English language. No date delimiter was included in the search criteria. In total, 42 studies from 1980 to 2019 were returned by the above search criteria. J.K.Y, P.S.U., and L.N.A. independently reviewed each article for inclusion. Study authors reviewed each article and determined its relevance to U.S. pediatric rural TBI/concussion. Articles were excluded if they studied a non-U.S. population, did not study TBI, did not provide data on pediatric TBI, or if they were review articles. All articles were unanimously included or excluded. 

**Figure 1:**
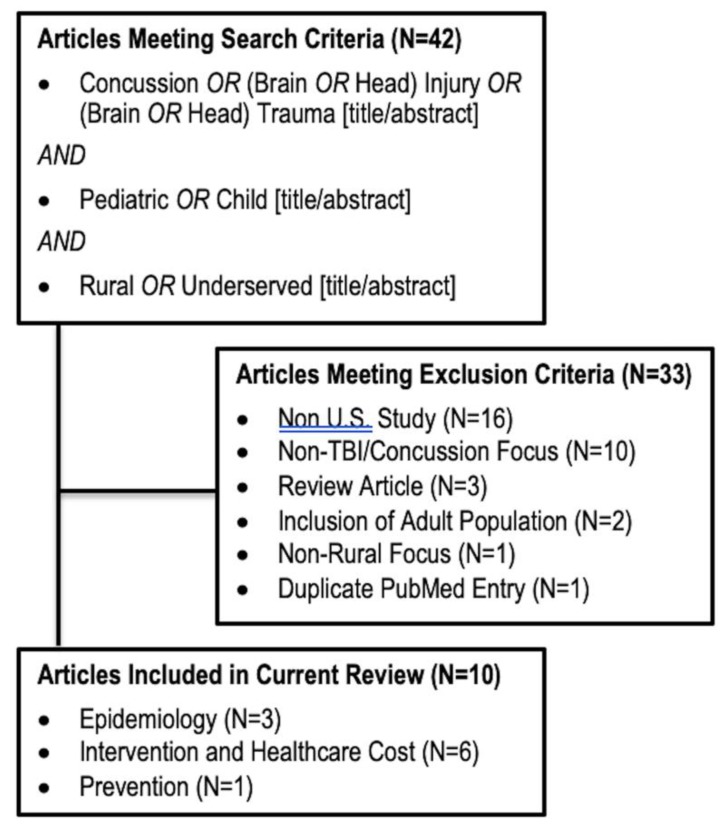
Flow Diagram of Included Articles.

Of the 42 articles from the initial search, 10 were selected for inclusion (Figure 1). Thirty-three articles were excluded for the following reasons: non-U.S. study (*N* = 16), non-TBI/concussion focus (*N* = 10), review article (*N* = 3), inclusion of adult population (*N* = 2), non-rural focus (*N* = 1), and duplicate PubMed entry (*N* = 1). 

### 2.2. Study Analyses

The included articles were stratified by subtopic, including epidemiology (*N* = 3), intervention and healthcare cost (*N* = 6), and injury prevention (*N* = 1). Each subtopic was synthesized to elucidate the issues unique to TBI/concussions in rural pediatric populations, with comparisons to urban populations when available.

## 3. Results

### 3.1. Epidemiology

Overall, the studies reported increased overall trauma and head injury severities in rural regions, with higher mortality and reduced access to Level I and II trauma care. Missing data was more prevalent in rural TBI. 

#### 3.1.1. Oregon Trauma Registry Study

Leonhard et al. reported on demographic and injury factors, and rates of morbidity and mortality in 2794 pediatric TBI patients, using retrospective data from the Oregon Trauma Registry from 2009 to 2012. By distribution, 47% were large metropolitan, 25% small/medium metropolitan, and 29% rural/non-metropolitan [16]. Rural children were more likely to be older (15–19 years: 51% vs. 46–47%) and have an unknown/missing race (16% vs. 3–5% across metropolitan regions). The statistical relationships toward the available level of care, decreased injury severity, and decreased missing data correlating with increasing population density were observed across rural, small/medium metropolitan, and large metropolitan regions as follows (all, *p* < 0.0001): Level I/II trauma center (rural: 45%, small-medium metropolitan: 69%, and large metropolitan: 96%), transfer to higher level of care (24%, 19%, and 2%), injury severity score (ISS) >15 (24%, 29%, and 34%), and missing insurance data (16%, 9%, and 2%). The small/medium metropolitan regions were similar to rural regions in motor vehicle collisions (36% and 37% vs. 28%) and missing GCS scores (23% and 18% vs. 8%).

Mortality was higher in rural regions at 5% vs. 2%. The authors performed a multivariable analysis controlling for the demographic and clinical factors, including age, gender, race, insurance status, injury severity, and blunt vs. penetrating TBI. Even after adjusting for these factors, the rural patients had a higher incidence of TBI (107 vs. 71 per 100,000) and also had a two-fold increase in mortality (OR (odds ratio) = 1.8, 95% CI (confidence interval) 1.0–3.3) [16]. The potential reasons for this disparity in mortality include longer transportation times (>30 min: 20% rural vs. 15% large metropolitan) and lack of treatment (no treatment in ED: 95% rural vs. 84% large metropolitan). The greater incidence of TBI in rural populations was reflected by an increase in adolescent TBI (ages 15–19). Other authors have shown that this increase is correlated with an increase in motor vehicle collisions in rural populations from multiple types of vehicles, including cars, ATVs, and water crafts [17]. These data reflect that despite higher injury severities, the comparative deficits in healthcare resources and coordinated care in rural areas are likely the culprits to poorer outcomes.

#### 3.1.2. Pediatric Health Information System Study

Loftus et al. reported on retrospective data from TBI due to unintentional falls in 23,813 children admitted to hospital from 2009 to 2014 using the Pediatric Health Information System (PHIS), which collected data across 35 participating U.S. hospitals [18]. Falls from a building accounted for 4% of all falls and constituted the group of interest. While TBIs due to falls from a building did not differ statistically between rural and urban settings, the rural rates of serious/severe TBI, as defined by the Abbreviated Injury Scale of the head (AIS) 3–6, were significantly higher (59% vs. 54%, *p* < 0.01). Rural children were more likely to suffer a skull fracture with extra-axial hemorrhage (37% vs. 29%; *p* = 0.03). No differences by setting were found for mortality (<1%) or treatment factors (ICU: 45% vs. 47%; operating room: 13% vs. 12%), including the level of care (Level I trauma center: 85% vs. 87%). For demographics, the rural patients were more likely to be Caucasian (21% vs. 52%, *p* < 0.01), non-Hispanic (10% vs. 18%, *p* < 0.01), and have lower rates of government insurance (45% vs. 61%, *p* < 0.01). The caveat of this study lies in the target cohort of TBI, specifically due to falls from a building. Nevertheless, a higher injury severity and rates of extra-axial hemorrhage in rural children may be proxies for differences in underlying injury mechanisms, protracted transport times, and care coordination at the treatment site.

#### 3.1.3. Minnesota Olmsted County Skull Fracture Study

Nelson et al. retrospectively reported on the skull fractures due to head trauma in a primarily rural region of Olmsted County, Minnesota, from 1935 to 1974 [19]. The incidence was 44.3 per 100,000 person–years and this rate remained unchanged during the final 30 years of the study. In those aged 0–5 years, there were more TBIs with than without fractures (males < 1 year-old: 260 vs. 50 per 100,000 person–years; males 1–4 years: 100 vs. 80 per 100,000 person–years), and comparatively, a dramatic decrease of TBI with a fracture compared to without a fracture in those aged 15–19 years (males: 100 vs. 450 per 100,000 person–years). Simple linear fractures predominated in pediatric injuries. The authors compared the incidence of skull fracture by age between Olmsted County and the primarily urban city of Rochester, Minnesota, and found that skull fractures were less in the former (38 vs. 49 per 100,000 person–years); comparatively, there were more rural TBIs due to MVA (Motor Vehicle Accident) (Olmsted vs. Rochester: 39% vs. 26%) and occupational injuries (8% vs. 4%), with less proportion of falls (25% vs. 42%). This supports the findings alluded to by Leonhard et al. that the increased incidence of rural pediatric TBI can be tied to the increased use of motorized vehicles and occupational injuries. Finally, without commenting on age, rural residents had more basilar (23% vs. 16%) and compound fractures (15% vs. 11%), which often occurred after high-speed MVAs, and less simple linear fractures (46% vs. 57%) [19].

### 3.2. Intervention and Healthcare Cost

Overall, rural pediatric TBIs incurred greater healthcare costs while having a decreased access to mental health and specialist resources. Rural patients were at increased risk of triage to non-trauma healthcare facilities, protracted transport times, and factors affecting transport duration—e.g., inclement weather. Less severe injuries were at a higher risk of delayed transport.

#### 3.2.1. MarketScan Insurance Claims Study: Utilization of Healthcare Services and Cost in Mild TBI

Graves et al. reported on 387,846 children with mTBI captured by the MarketScan Commercial Claims and Encounters database, which includes healthcare utilization and cost data for over 20 million U.S. employees and dependents with private/commercial insurance [20]. The subjects had ≥ 1 TBIs by the International Classification of Diseases, Ninth Revision, Clinical Modification (ICD-9-CM) codes between 2007–2011. The rural patients accounted for 13% of the sample and required higher comparative healthcare costs in the 180 days after the mTBI, with an adjusted cost ratio of 1:11 (95% CI 1.06–1.16). While both rural and urban patients had a similar raw exposure to physical and occupational therapy (PT/OT, 3.2% vs. 3.5%, respectively), after adjusting for covariates, the rural children had 15% increased utilization of PT/OT and 51% more costs compared to urban children. Conversely, rural children utilized less mental health services (4.3% vs. 5.5%), at 63% that of urban patients, with 74% of costs after adjusting for covariates.

Notably, rural children had lower baseline healthcare costs in the 180 days prior to the TBI ($278 vs. $345) but required higher costs in the 180 days post-TBI ($2871 vs. $2479). The authors discussed that reasons for decreased mental healthcare utilization may include stigma, lower rural access to advanced healthcare resources, as well as misclassification of mental health as general health appointments. Regarding the increased PT/OT utilization, these rehabilitation interventions may be more familiar to rural patients, who may also suffer more severe injuries, as shown in other studies. 

#### 3.2.2. North Dakota Emergency Medical Services Study: Suboptimal Triage to Care Facility in Moderate to Severe TBI

The population density of North Dakota is 9.3 people per square mile, with 25% under 18 years of age, and 5% Native American in origin. At the time of study, North Dakota had no Level I and only six Level II trauma centers. Accordingly, most patients were triaged in Level IV hospitals that required a higher level of efficiency in triage and transfer mechanisms, which was not always available. Poltavski et al. retrospectively examined North Dakota’s emergency medical service (EMS) ambulance records from 1995 to 2000 to identify patients aged 0–18 years, who were transported to a non-designated acute care facility rather than a designated trauma center (Level II–V) [21]. In 156 pediatric TBIs with a GCS of 3–12 from rural settings, predictors to inappropriate triage were Native American (OR = 16.1), distance to trauma center (OR = 1.2, distance not stated but likely per-mile), and winter vs. other seasons (OR = 9.3). The authors concluded that geographic and weather conditions, in addition to ethnic considerations, such as injury occurring on a Native American reservation, which is more likely to be further from a care facility, must be considered in healthcare triage and coordination decisions [21], especially in the construction of new roads or healthcare facilities. This finding also highlights the reduced resource allocation toward underrepresented groups in trauma healthcare.

#### 3.2.3. Utah Trauma Performance Measures Study: Predictors of Suboptimal Triage and Transfer in Severe Trauma

Gleich et al. evaluated the predictors of prolonged triage or transfer; prolonged triage was defined as greater than 2 h from presentation, while prolonged transfer was defined as greater than 6 h for rural patients. The authors studied 412 severe pediatric TBI patients (injury severity scale score (ISS) > 15) who were transferred to the accepting Level I trauma center Primary Children’s Medical Center (PCMC) in Salt Lake City, Utah. A majority of patients (72%) had a documented head/neck injury [22]. Fifty-four percent were triaged within 2 h, and those who were triaged within the goal had more severe overall trauma (median ISS 20 vs. 17), more severe brain injury (GCS 3–8: 36% vs. 17%), were more likely to be victims of abuse (19% vs. 9%), and had a greater distance to PCMC (median 68 vs. 150 miles). The predictors for delayed triage included less severe overall head trauma, as well as a greater distance to PCMC. Accordingly, children who were triaged within 2 h were more likely to require ICU admission, and had a five-fold increase in mortality (10% vs. 2%). Interestingly, 94% of rural transfer times were within the goal, compared to 76% of urban transfers. The authors concluded that there was significant non-adherence to the performance standard of “within 2 h” among severe pediatric trauma patients, for which quality metrics should be established [22]. 

#### 3.2.4. Iowa Emergency Department Transfer Study: Potentially Avoidable ED Transfers in TBI

Mohr et al. retrospectively analyzed the Iowa Hospital Association ED transfers from 2004 to 2013 to identify potentially avoidable transfers (PATs). PATs were defined as patients who were transferred to another hospital and either discharged from the ED or admitted for less than 24 h [23]. Of 2,117,317 total pediatric ED visits, 1% were transferred. Thirty-nine percent of transfers were deemed PATs, of which 66% were from rural regions, associated with a $909 cost increase. In the 17,066 TBIs from the dataset, 2.5% were transfers, of which 48% were PATs associated with a $1455 cost increase. TBIs constituted the diagnosis with the highest increase in cost for PATs across all diagnoses. While the authors did not substratify the TBI costs by urban vs. rural, the work reflects that transfers due to a perceived lack of resources and regional healthcare inequities may compound inefficiencies and costs in care, with unknown effects on the outcomes [23].

#### 3.2.5. Texas Urban vs. Rural Designation Study: Injury Severity and Outcomes in Severe TBI

Robertson et al. retrospectively analyzed 444 patients from the Children’s Medical Center Dallas, which receives patients from the states of Texas, Oklahoma, and Arkansas [24]. Patients were either traditionally classified into “large” or “small towns” or classified based on zip code using a Rural and Urban Commuting Area Code (RUCA2). The authors hypothesized that rural versus urban designations at the county level may be inaccurate, as urban designations often include small rural towns distant from tertiary pediatric trauma centers. The authors posited that zip code classifications (e.g., RUCA2) were more appropriate for urban/rural classifications. 

The mean age was 5.7 years and the discrepancy was 9% rural under the traditional classification vs. 18% rural under RUCA2. Within both classification systems, rural and small-town TBI patients had higher injury severity scores (ISS; 17 vs. 15, 18 vs. 15, respectively), and longer hospital stays (7 vs. 5 days, 9 vs. 5 days, respectively), compared to urban or large-towns, while the initial GCS (scene: 5.2–5.3, ED arrival 3.6–3.7) were comparable. This study further shows that under certain classification schemes, urban areas may include remote locales which deserve reclassification, of which clinicians should be aware in order to improve healthcare delivery to specific subpopulations [24]. This further highlights an issue with resource allocation, as oftentimes, grants are apportioned to counties, not zip codes. Targeted interventions to the rural zip codes are important to ameliorate these health disparities. 

#### 3.2.6. Oregon Parental Focus Group Study: Survivor Impact after Neurocritical Care

Williams et al. prospectively analyzed survivor symptomatology after injuries requiring neurological ICU care, including post-intensive care syndrome (PICS)—a constellation of patient morbidities, including physical, cognitive, emotional, and psychological impairments following ICU care [25]. The focus groups from one children’s hospital in Oregon were conducted with 16 parents of rural pediatric neurological injury. Sixty-seven percent of children suffered a TBI, and the remainder strokes and meningitis/encephalitis. Eighty-nine percent were aged 0–8 years. Major findings included: pediatric neurocritical care was intensely emotional for family and survivors, a large proportion lived with chronic illness (89% require ongoing medical attention, 33% psychological/counseling services, 56% at-school services), limited access to primary and specialty care (including mental health) in rural settings, and the need for education and awareness of PICS and healthcare follow-ups for children with neurological injuries, especially in rural communities. Parental perspectives should also be considered by clinicians and researchers after TBI.

#### 3.2.7. Georgia Bicycle Helmet Study: Pre- and Post-Mandate Adherence

Gilchrist et al. reported on the incidence of pediatric helmet use in rural communities before and after a statewide mandate for helmet use in all riders <16 years of age using a prospective observational study. Rather than instituting a fine, police impounded the bicycle until helmet ownership was verified. The authors found that helmet use improved from a pre-mandate of 0% to 30–71% within 5 months-post-mandate in children of 5–12 years of age across race and sex cohorts, while the improvement was modest in teens of 13–15 years (0% to 0–50%) and negligible in adults and teens >15 years (0% to 0–6%).

Nearly 28 million U.S. children <15 years of age ride bicycles [26]. In 1997, nearly 367,700 children sought ED care for bicycle-related injuries, of which, 30% were head injuries [27]. Studies have shown that 69–88% of serious TBIs related to bicycles can be prevented with helmet use; however, studies also show that only half of children own a helmet and one-quarter use helmets every time they ride [26]. The authors concluded that active, continued enforcement with education and awareness promotion could lead to lasting changes in prevention [26].

### 3.3. Included Studies

The included studies of the current review are displayed in the following Table 1.

## 4. Discussion

Distinctions between rural and urban pediatric TBI center on several key factors. Rural pediatric TBI patients generally present with higher injury severity and a lower GCS. The overall costs incurred are greater due to longer hospital stays, transfers to larger institutions, and decreased availability of specialist care. Understanding the differences in patient presentation and resource utilization is critical for improving the outcomes for this vulnerable population. We synthesized these clinical characteristics and associations in order to highlight the current gaps in care and aid in the creation of the best practices for pediatric TBIs across rural/underserved care settings.

### 4.1. Epidemiology

Overall, studies reported increased overall trauma and head injury severities in rural regions, with higher mortality and reduced access to Level I and II trauma care. Multiple studies showed increased injury severity scores and decreased GCS in rural TBIs. These studies highlight the need for primary prevention and education efforts using public policy initiatives and enforcement [26,27]. It is also possible that only more severe and/or symptomatic TBI cases are evaluated in rural clinics or hospitals, due to obstacles to the access of care. The finding of the underreporting of TBI in rural settings further compounds the fact that rural populations have higher TBI incidence, as described by Leonhard et al. [16]. Studies have shown that pediatric TBI data is difficult to collect, and often missing from hospital registries or medical records [16]. Coupled with potential underreporting, the need for complete data for rural population statistics becomes even more relevant. It is important for clinicians and healthcare institutions in rural/underserved settings to be cognizant of these issues, and work with multidisciplinary healthcare staff to improve the completeness of clinical data collection at the time of injury, during the hospital course, and at follow-up time points. National efforts for the standardization of relevant data collection, such as the National Institutes of Health Common Data Elements for Traumatic Brain Injury, have received validation in practice utility for the pediatric population over the past decade and are readily adoptable [28,29]. With a complete clinical record and high-quality data, clinicians, institutions, and researchers will be able to better understand the current state of care, identify treatment and practice patterns, and consolidate data across multiple institutions to generate conclusive and actionable data.

### 4.2. Intervention and Healthcare Cost

Rural pediatric TBIs incurred greater costs while having a decreased access to mental health and specialist resources. Moreover, rural patients were at an increased risk of triage to non-trauma facilities while facing protracted transport times [21]. Injury severity inversely correlated with time to transport. These findings highlight the need for evidence-based protocols and triage guidelines for efficient clinical decision-making. Moreover, standardized guidelines for stratifying mTBI patients beyond the GCS classification could help direct resources to patients in need. Rural pediatric TBI patients are at higher risk of TBI and the historical triage systems may be inadequate in improving mortality and long-term outcomes [22,24]. Rural patients may have less severe TBI but be transferred inappropriately to other hospitals, thus, incurring avoidable costs. Rural settings are also at risk of the suboptimal triage of moderate and severe TBI compounded by delays in care [20,21]. The increased costs to patients explain, in part, the lack of engagement with follow-up care. The insurance paradigms available to rural children were not explored in detail in existing studies and should be incorporated into future studies. Addressing these issues will not only require changes in practice guidelines, but also investment into rural communities for healthcare facilities and healthcare providers on national and local levels across public and private sectors. 

Perhaps most detrimental for the long-term outcomes of rural pediatric TBI patients is the decreased utilization of mental health services [20]. Psychiatric sequelae following TBI are prevalent, well-documented, and can profoundly and permanently impact the patient’s quality of life and economic productivity [30,31,32]. The development of PTSD and major depressive disorder are also linked to demographic risk factors, such as trauma secondary to violence, and lower education levels [30]. Given the overlap between the rural population and these risk factors, rural pediatric TBI patients may be most at risk for the development of secondary psychiatric symptoms. In fact, non-hospitalized TBI patients are shown to have high rates of anxiety and depression and often require specialized neurological and psychiatric services [33]. Barriers to the access of care often compound these issues in rural patient populations and constitute a critical area for the attention of institutional leadership and consultants.

Telemedicine services may provide a solution to decrease the time to triage, while also decreasing the cost for rural patients. Numerous other countries have utilized telemedicine triage services to increase the accessibility of neurosurgical care. Doing so can decrease unnecessary transport while providing high quality specialized care at a fraction of the cost [13]. Finally, as documented by Graves et al., rural patients utilize PT/OT services at a higher rate following TBI. Broadening the scope of PT/OT to include mental health services and neuropsychiatric evaluation can help reach this vulnerable patient population.

### 4.3. Prevention

Prevention efforts can be successful in rural regions, as evidenced by the Georgia Bicycle Helmet Study. Three features of this case can provide a model, by which other locales can have effective targeted policy initiatives [27]. First, the at-risk patient population of children who did not wear helmets while biking was identified. Second, a multi-tiered approach focusing on awareness and education along with enforcement through local law enforcement was established and supported [27]. Finally, the policy enforcement was constructive and educational rather than legally punitive, which improved compliance and policy awareness in parents, children, and society without creating resentment. This model should be applied to other at-risk populations—e.g., motor vehicle accidents and child abuse cases [16,22,27].

### 4.4. Limitations

A few key limitations exist within the literature. Rural areas have unique regional features that limit the generalizability of the findings described. This is compounded by the small number of studies that have examined pediatric TBI in rural/underserved populations. The lack of prospective studies that evaluate outcomes make the creation of guidelines difficult. Context-specific best practices are needed and should be focused upon in future studies. We focused on peer-reviewed literature, available broadly in the PubMed database, and did not systematically explore other databases, such as the Cumulative Index to Nursing and Allied Health Literature (CINAHL) and PsycINFO (American Psychological Association), which limits the breadth of related studies included in this review. We also focused on U.S. studies and practices. An internationally-focused systematic review on rural pediatric TBI will address these limitations, which we intend to undertake in the future. 

## 5. Conclusions

U.S. rural pediatric patients are at higher risk of dangerous injury mechanisms, trauma severity, and TBI severity compared to urban. The barriers to care include protracted transport times, transfer to less-resourced centers, increased healthcare costs, missing data, and decreased access to mental health and/or specialty care during hospitalization and follow-up. Preventative efforts can be successful, and will require improved multidisciplinary awareness and education across multiple healthcare and government agencies.

## Figures and Tables

**Table 1:** Summary of studies.

***Epidemiological Studies***				
**Author**	**Study Type**	**Description**	**Results**	**Conclusions**
Leonhard et al. 2015	Retrospective cohort study	2794 pediatric TBI cases in rural vs. metropolitan areas abstracted from Oregon Trauma Registry 2009–2012.	Rural children had higher annualized rates of TBI incidence than metropolitan children (107 vs. 71 per 100,000) and a 2-fold increase in odds of mortality following TBI (OR 1.8, 1.04–3.3) adjusting for demographic factors.	Rural patients have greater TBI incidence and worse outcomes. Missing data is a pervasive problem for rural cases.
Loftus et al. 2010	Retrospective cohort study	23,813 pediatric hospitalization abstracted from Pediatric Health Information systems between 2009–2014.	Rural children sustaining building falls were more likely to have serious/severe TBI compared to urban children (58.9% vs. 53.6%; *p* < 0.01)	Rural children sustaining TBI due to falls sustain more severe injuries than urban children.
Nelson et al. 1984	Retrospective cohort study	3598 head trauma cases reported from rural Olmsted County, Minnesota	Higher incidence of TBI caused by MVA (39% vs. 26%) or occupational injury (8% vs. 4%) as compared to Urban Rochester MN. Younger patients (ages 0–5) had greater incidence of concomitant fracture with TBI.	Younger children were more likely to suffer complex TBI. MVA and occupational injuries are the most prominent mechanisms of injury.
***Intervention and Healthcare Cost***				
**Author**	**Study Type**	**Description**	**Results**	**Conclusions**
Gleich et al. 2011	Retrospective cohort study	412 patients abstracted from Primary Children’s Medical Center (PCMC) database from 2006 to 2009.	50% of pediatric TBI cases were transferred and triaged from original hospital in <2 h. Factors associated with delayed triage included: less severe head injury, greater distance from hospital, primary chest/abdominal injuries. Outcomes did not differ between patients transferred <2 h and those transferred >2 h.	Patients sustaining severe head injuries were more likely to be transferred within the 2-hour practice guideline. The effect this has on outcomes is unclear.
Graves et al. 2019	Retrospective cohort study	387,846 mild TBI cases abstracted from MarketScan Commercial Claims and Encounters Data 2007–2011	Healthcare costs for pediatric mild TBI was $2778 for rural patients and $2499 for urban patients (*p* < 0.01). Urban children utilized more speech therapy and mental health services than rural children. Rural children utilized more PT/OT.	TBI costs are greater for rural patients. Rural patients use of more PT/OT services but less mental health and speech therapy services requires further examination.
Mohr et al. 2016	Retrospective cohort study	2,117,317 rural pediatric ED admissions between 2004–2013 were assessed for potentially avoidable transfers.	Isolated traumatic brain injury without extra-axial bleeding was frequently identified as a potentially avoidable transfer and incurred an additional cost of $1455 to patients.	Creating best practice guidelines can minimize cost to patient and the healthcare system while maintaining or improving outcomes.
Poltavski et al. 2005	Retrospective cohort study	156 cases of pediatric head injuries with GCS ≤ 12 in North Dakota from 1995 to 2000.	Mistriage, defined as transportation of pediatric patients with moderate to severe TBI to a non-trauma center occurred more frequently in winter and if the child was Native American. Distance to trauma center and shorter distance to receiving facility also corresponded with rates of mistriage.	Distance and race are two key issues that should be addressed in policy aimed at optimizing triage of pediatric TBI patients.
Robertson et al. 2011	Retrospective cohort study	444 patients treated at Children’s Medical Center Dallas classified into urban city, large town, small town, or isolated town based off of commuting area codes	Isolated town TBI patients had higher injury severity scores (ISS; 17 vs. 15), and longer hospital stays (7 vs. 5 days) than urban city patients.	Rural pediatric TBI are generally more severe than urban areas.
Williams et al. 2018	Focus group study	16 parent caretakers of pediatric patients with TBI or other neurologic insults assessed in 4 focus group sessions.	Pediatric neurocritical care has an immense and long-lasting effect on families and survivors. 89% of patients require ongoing medical attention, 33% require psychological counseling and 56% require at-school aid. Rural settings have limited resources compounding financial burdens for families.	Outcome optimization for pediatric TBI patients requires long-term support for survivors and their families
***Prevention***				
**Author**	**Study Type**	**Description**	**Results**	**Conclusions**
Gilchrist et al. 2000	Prospective observational study	In April 1997, 580 students from kindergarten to 7th grade received bicycle helmets and education and were observationally followed.	Observed helmet use rose from 0% pre-intervention to 45% post-intervention. Police impounded bikes of helmetless riders. Two-years after study initiation helmet use was observed at 54%	Education and enforcement can create behavioral changes that can mitigate pediatric TBI.

TBI = Traumatic brain injury; MVA = Motor vehicle accident; PT/OT = Physical therapy/occupational therapy; ED = Emergency department; GCS = Glasgow Coma Scale.
